# Treatment of Pneumonitis, Etc.

**Published:** 1852-02

**Authors:** 


### EDITORIAL DEPARTMENT.

*Treatment of Pneumonitis, etc.* — In the Western Lancet, No. for Dec., 1851, we find the following statement of certain statistical results bearing on the management of pneumonitis: —

"An Austrian physician (Dr. Dietl) has published the results of different methods of treating pneumonia; and it appears that he was quite liberal in his views, and at times adopted widely different methods of treatment, ranging from blood-letting to homœopathic *nothingness.* Dr. Dietl gives the following results: Of 85 cases treated by bleeding, 17 (or 1 in 5) died; of 106 treated by tartar emetic, (without bleeding) 22 (or 1 in 4.08) died; of 189 treated by a purely *expectante* method, 14 (or 1 in 13.5) died. The mortality under the homœopathic treatment was exactly the same as under the *expectante* method."

The able editor of the Lancet comments on the indefiniteness of such statistics. He says, what is obvious, that modifying or controlling circumstances which are wholly left out of the calculations, may have determined the diversity of results under the different methods of treatment. "Were the patients," he asks, "old or young — ill or well fed — confined to a vitiated or pure atmosphere, and, finally, was the disease sthenic or asthenic?" "Were the observations drawn from epidemic or sporadic cases?" Questions, like these, suggest sources of error in the application of statistical comparisons to therapeutics, which are with difficulty obviated, and which lead us to distrust the numerical method as a means of advancing our progress in practical medicine.

In one point of view, however, the foregoing statistics are valuable, as the Editor of the Lancet remarks. They show, to quote the writer's language, "that infinitesimal doses and *nothing* are, in a therapeutical sense, exactly synonymous." The mortality under the homœopathic treatment and the expectant system was similar. This is what would be expected, denoting doubtless more than merely a singular coincidence.

In another point of view the statistics appear to us to possess considerable value — a point of view not noticed in the remarks of our valued contemporary. The ratio of recoveries under the expectant method exceeded, by more than double, that under the treatment by bleeding, and was trebly greater than that under the treatment by tartar emetic. Now, although these facts do by no means prove that bleeding and tartar emetic are not useful remedies in the treatment of certain cases of pneumonitis, they do lead to an important practical conclusion, viz., that a very large proportion of cases of this disease do well without active medicinal interference. This conclusion must be admitted, assuming that the statistics are correct, for which we cannot, of course, vouch. This, we say, is an important practical conclusion. Considered in connection with the results in the cases treated by bleeding and tartar emetic, it is fair to infer that it is vastly better to withhold these remedies entirely in the treatment of this disease, than to resort to them indiscriminately. This leads us to a fundamental truth in practical medicine, the importance of which can hardly be over estimated, and which, there is reason to fear is not so generally and fully appreciated as it ought to be. The truth to which we allude is, that the efficacy of powerful remedial agencies in the management of diseases, depends on the discrimination with which they are employed. A bleeding in pneumonitis may be necessary to the safety of one patient, while it takes away all chance of recovery in another. So with all acute affections, and the various measures of therapeutics. The power which renders them efficiently curative in one instance, causes their operation to be not less destructive in another. The armamenteria medica cannot be wielded without effects for evil, or for good. Success in the practice of medicine, therefore, depends entirely on the judgment of the practitioner in directing the resources of the art.

The advancement made in practical medicine of late years, consists, in a great measure, in the greater discrimination and discretion observed in the employment of active remedies. This has resulted from progress in knowledge of pathology, and of the natural history of diseases, together with a better understanding of what belongs to true medical experience. It is not a correct inference from the fact of bleeding, and other potent agencies being less employed than formerly, that their real value has undergone a depreciation. Their use has been restricted to the cases in which they may be resorted to with safety and advantage, but, thus limited, their efficacy is even more conspicuous at the present moment than at any previous period in the history of medicine. The contrast in the modes of practice pursued by different members of the profession is more marked in the discrimination regulating active medicinal interference than in any other point of view. There is a class of practitioners who act on the principle that every malady is to be treated by vigorous means, which are to be diligently applied until death or recovery, without reference to the self-limited character or intrinsic tendency to a favorable issue which belongs to numerous diseases. Physicians of this stamp generally are accustomed also to direct their treatment after stereotyped rules, applied indiscriminately to all cases, and under every diversity of circumstances. A patient with pneumonitis must be bled and take tartar emetic, as a matter of course, and so with other measures in other affections. Thanks to the progress of science, this class is yearly becoming less and less, and in proportion as the profession is pervaded by the influence of more rational views deduced from pathology and confirmed by experience, will the beneficent effects of our art be enhanced, and its character rise in public estimation.

It has been customary to apply the phrase, *heroic practice* to boldness in the use of remedial agencies. The expression is a significant and just one. It certainly denotes a species of courage to meet disease promptly, and vanquish it by a successful onslaught. But there is another phase of heroism in medical practice, in which the conduct of the practitioner claims not less our admiration. This consists in the boldness of withstanding the temptations from within, and perchance solicitations from without, for active interference, whenever the indications for potent remedies are not present, notwithstanding the dangerous condition of the patient, knowing that unless the powerful resources of the materia medica are indicated, they cannot fail to do harm. This kind of courage is, in some measure, a characteristic of modern medicine. It is to the physician what "masterly inactivity" sometimes is to the military commander. The latter is less attractive and brilliant than feats of daring which only require a physical indifference to danger, but it demands more science, greater self-reliance, and often denotes the truly able tactician.

To revert, after this digression, to the treatment of pneumonitis, we are at no loss to understand, that in a series of cases in which the bleeding and tartar emetic treatment were *indiscriminately* and *efficiently* pursued, the rate of mortality should be greater than in a series of cases in which these remedies were omitted. They are useful, often highly so, in the management of this disease; but this is very different from saying that they are uniformly called for, or will not oftener do harm than good if not directed with discretion. The existence of pneumonitis does not involve the necessity of these, or any other active remedies.* They are not to be resorted to simply because a patient has pneumonitis, but when the disease is accompanied by circumstances which indicate their propriety. To consider these circumstances

---

* We have treated, with very slight medication, cases of this disease which we may report at some future time.

would lead us into a field of discussion which it is entirely foreign to our present purpose to enter upon. The train of reflection we have indulged has been casually suggested by the extract from the Western Lancet upon which, when we began to write, we did not intend to make more than one or two brief comments. We venture, however, to hope that our remarks may not be wholly useless if they should chance to prove suggestive to the minds of any of our readers who need a hint on the score of the *nimia diligentia medicorum.*

